# Endothelin-1 and Its Role in Cancer and Potential Therapeutic Opportunities

**DOI:** 10.3390/biomedicines12030511

**Published:** 2024-02-23

**Authors:** Madeline Harrison, Dmitry Zinovkin, Md Zahidul Islam Pranjol

**Affiliations:** 1 Department of Biochemistry, School of Life Sciences, University of Sussex, Brighton BN1 9QU, UK; meh29@sussex.ac.uk; 2 Department of Pathology, Gomel State Medical University, 246000 Gomel, Belarus; pat_anatomy@gsmu.by

**Keywords:** endothelin-1, angiogenesis, metastasis, migration, drug resistance, therapeutics

## Abstract

Endothelin-1 (ET-1) plays a physiological role as a potent vasoconstrictor. It is implicated in an array of diseases, and its signalling is often found to be overactivated within cancers. ET-1 has been found to potentiate hallmarks of cancer progression such as cell proliferation, invasion and metastasis, as well as angiogenesis. ET-1 has also been implicated in inducing the epithelial–mesenchymal transition (EMT) and promoting resistance to anticancer drugs. Many preclinical efforts have been made to target ET-1 expression within cancer, such as by using ET-1 receptor antagonists, many of which have been approved for treating pulmonary hypertension. Targeting ET-1 has been shown to improve the response to various other cancer therapeutics, highlighting the potential benefits targeting this peptide may exert. Drug repurposing is an attractive strategy, and exploration of this avenue may be promising for targeting ET-1 in cancer. There are many clinical trials which have been completed and are currently undergoing involving the repurposing of ET-1 receptor antagonists for cancer treatment. In this review, the pathways through which ET-1 potentiates cancer will be discussed, as well as where the opportunity for therapeutic intervention lies in relation to cancer.

## 1. Introduction

Cancer is one of the most prevalent diseases globally. In 2008, approximately 12.7 million cancer cases and 7.6 million cancer deaths were reported [1]. In 2020, approximately 19.3 million new cancer cases were documented (52% increase), with 10 million cancer-related deaths recorded worldwide (a 32% increase compared to 2008) [2]. It is evident that cancer incidence rates are on the rise, with a predicted 47% increase in cases by the year 2040, equating to 28.3 million new cases [2]. In addition, early-onset cancer has seen a 79.1% increase between 1990 and 2019, meaning the incidence rates amongst younger populations are increasing [3].

To tackle rising cancer rates, more selective and specific treatment approaches are being tested alongside the existing traditional cancer treatments such as chemotherapy and radiation therapy, along with surgery. Despite this, many cancer targets are not well defined, with chemotherapy often remaining the most effective approach [4]. However, chemoresistance is a challenge in therapeutics, with many cancer cells escaping drug response, resulting in a plateau in chemotherapy success [4,5]. Another problem posed in cancer treatment relates to the high toxicity associated with chemotherapy treatment, resulting in unpleasant side effects for patients [4]. Consequently, the need to develop more targeted therapeutics is becoming more crucial.

Endothelin-1 (ET-1) is a potent vasoconstrictor which has been implicated in many cancer-promoting mechanisms [6,7]. Thus, currently, ET-1 is being investigated in anticancer drug development. In this review, the role of ET-1 in potentiating cancer will be examined. In addition, we aim to explore the therapeutic potential of ET-1 in cancers.

## 2. Biology of Endothelin-1 (ET-1)

ET-1, a 21-amino-acid peptide belonging to the endothelin (ET) family, was first discovered in 1988 by Yanagisawa et al. [6]. ET-1 belongs to the ET-1 axis along with the G-coupled protein receptors (GPCRs), ETA receptor (ETAR) and ETB receptor (ETBR) [7]. One physiological role of the mitogen ET-1 is regulating basal vascular tone and vasoconstriction by acting directly on the smooth muscle cells [6,8]. Due to the ET-1 signalling through the GPCRs, there is potential for cross-talk between multiple signalling pathways; thus, the downstream effects of ET-1 signalling give rise to many functions of ET-1 [8]. For example, the processes that ET-1 has been implicated in include cell proliferation, angiogenesis, apoptosis regulation, metastasis and inflammation through the involvement of immune cell recruitment [7,8,9]. ET-1 has been shown to have a role in solid tumour progression but is also involved in other diseases such as cardiovascular diseases (CVD), including atherosclerosis, heart failure and hypertension, as well as asthma, and has been correlated with pain management [8].

**Figure 1:**
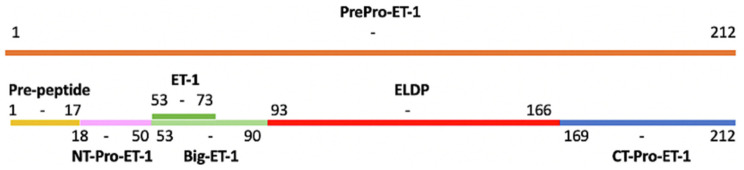
Peptide fragments of prepro-ET-1. Taken from Boutin et al. (2023) [11].

Encoded by the *EDN1* gene on chromosome 6, ET-1 synthesis initiates with the translation of its precursor, preproET-1. Inactive big-ET-1 is produced according to the proteolytic cleavage of prepro-ET-1, with intermediate proET-1 peptide formation, through the action of a signal peptidase and a proprotein convertase, often of the furin type [8,10,11]. The cleavage by endothelin-converting enzyme (ECE) of big-ET-1 produces the ligand ET-1, with the peptide fragments exhibited in Figure 1 [8,11]. *EDN1* gene activation occurs in response to certain physical or environmental stimuli. For example, hypoxic conditions, angiotensin II, cytokines, shear stress, insulin, growth factors and ischaemia, resulting in the release of ET-1 from the smooth muscle cells and endothelial cells of the vasculature and signalling in an autocrine or paracrine manner [12,13].

Although ET-1 is a multifunctional peptide, its mechanism of action is not fully understood. There is evidence for ET-1 as a therapeutic target for diseases such as late-stage cancer, where the current treatments may no longer be effective. Interestingly, the expression of ET-1 has been observed to be higher in many cancer cell types, specifically in ovarian carcinoma and in colorectal carcinoma, which is an incentive to investigate the underlying mechanisms of the role ET-1 plays in mediating tumour progression [12,14].

## 3. Role of ET-1 in Cancer Progression

**Figure 2:**
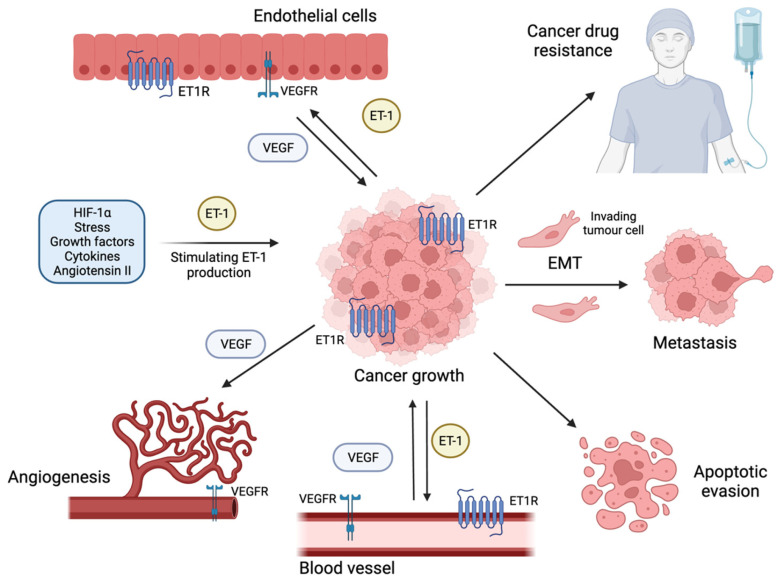
The role of ET-1 in cancer progression. ET-1 can be released from structures such as endothelial cells and smooth muscle cells. ET-1 receptors (ET1Rs) are often upregulated in various cancers. They are expressed on tumour cells, as well as endothelial cells, and can secrete ET-1 to form a self-amplifying loop. ET-1 production can be induced by an array of stimuli such as hypoxia, growth factors, stress, angiotensin II and various cytokines. ET-1 production can drive transcriptional changes to potentiate cancer growth. ET-1 promotes the epithelial–mesenchymal transition (EMT) to induce the metastatic phenotype, as well as promoting angiogenesis through ET-1-induced vascular endothelial growth factor (VEGF) production from the tumour. ET-1 has been shown to promote apoptotic evasion, as well as resistance to cancer drugs such as chemotherapeutics.

ET-1 has been shown to potentiate cancer by signalling through both ETAR and ETBR, where tumour progression is mediated via differential mechanisms. β-arrestin 1 (β-arr1) is a protein that is expressed in most tissues and was originally shown to negatively regulate GPCR signalling through receptor endocytosis. It is now defined to have many purposes, such as being a cytoplasmic scaffold for signalling components, as well as mediating signal transduction in the GPCRs, influencing many downstream processes [15,16]. β-arr1 is crucial to the regulation of many signalling pathways, and the dysregulation of these pathways is implicated in various diseases, including cancer. β-arr1, in the context of ET-1, mediates processes such as cell proliferation to promote tumour growth, metastatic behaviour, angiogenesis and chemoresistance. β-arr1 has been shown to permit crosstalk between multiple pathways downstream of the ET-1 axis, acting as a convergence point for tumorigenic signalling pathways [7,16,17,18,19,20]. Within this section, we will explore how ET-1 signalling affects different hallmarks of cancer progression (Figure 2).

### 3.1. Tumourigenesis and Metastasis

**Figure 3:**
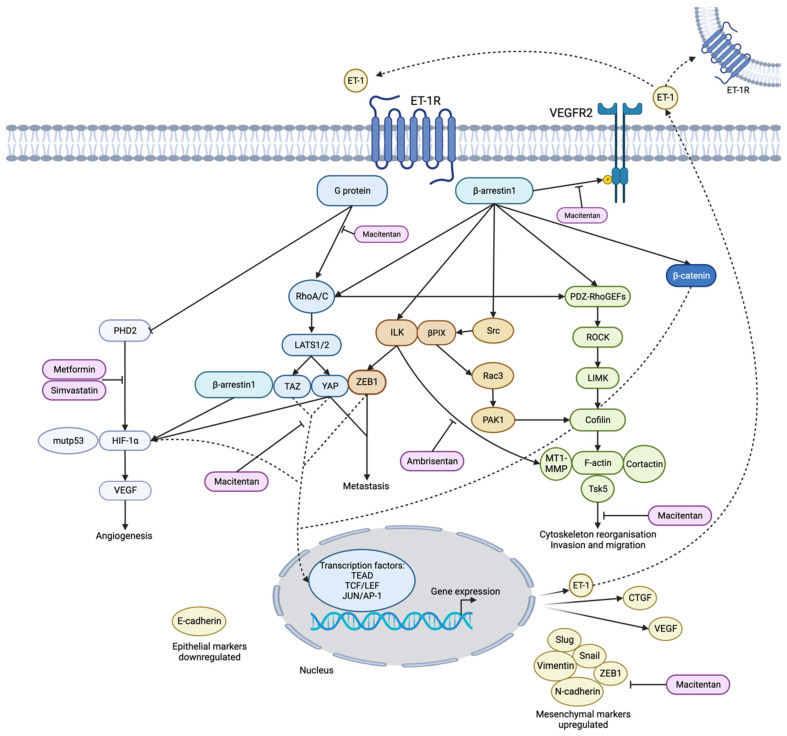
An overview of ET-1 signalling axis. ET-1 will signal through ETAR and ETBR (GPCRs) to activate a multitude of signalling pathways. The scaffold protein β-arrestin1 (β-arr1) facilitates the crosstalk between many signalling pathways downstream of ET-1 receptor activation. RhoA/C is upregulated upon ET-1 signalling, which inhibits the LATS1/2 kinases. Inactive LATS1/2 results in the dephosphorylation of Yes-associated protein (YAP) and TAZ, which permits their nuclear localisation. Association of YAP with TAZ induced by ET-1 is said to be cell-type-specific; found in colorectal carcinoma and high-grade serous ovarian carcinoma (HG-SOC) cells. Macitentan (ET-1 receptor antagonist) has been shown to block this nuclear localisation. Zinc-finger E-box-binding homeobox 1 (ZEB1) also forms an axis with YAP, which localises to the nucleus, where it interacts with the TEA domain (TEAD) transcription factor family and JUN (forms part of the AP-1 transcription factor). ZEB1 nuclear localisation is also assisted by integrin-linked kinase (ILK). ILK associates with βPIX, which is activated by Src, which subsequently activates Rac3. Rac3 phosphorylates and activates PAK1, which feeds into cofilin phosphorylation and actin polymerisation. Membrane-type 1 matrix metalloproteinase (MT1-MMP) is a direct substrate of ILK, and this regulation was blocked upon ambrisentan addition (ET-1 receptor antagonist). RhoC also associates with β-arr1, with PDZ-RhoGEFs induced by the ET-1 axis to activate LIM kinase (LIMK) and Rho-associated coiled-coil-forming kinase (ROCK), resulting in cofilin phosphorylation. Actin regulators are subsequently recruited to polymerised F-actin. Macitentan treatment has been shown to reduce the metastatic phenotype induced by this pathway. ET-1 inhibits enzymatic action of prolyl hydroxylase domain 2 (PHD2), promoting hypoxia-inducible factor-1α (HIF-1α) expression and vascular endothelial growth factor (VEGF) production. Metformin and simvastatin have been shown to upregulate the PHD2 enzyme to promote HIF-1α degradation. ET-1 can further promote the phosphorylation of VEGF receptor 2 (VEGFR2), but macitentan prevents this. YAP contributes to the stabilisation of HIF-1α downstream of ET-1. β-arr1 and β-catenin nuclear localisation was upregulated to target TCF/LEF transcription factors. Mesenchymal markers such as Slug, vimentin, Snail, ZEB1 and N-cadherin were upregulated coinciding with the downregulation of E-cadherin. ET-1 signalling upregulates its own production, which can form a positive autocrine feedback loop, but ET-1 can also signal in a paracrine manner.

Studies have outlined the effects ET-1 signalling can have in the context of tumour growth and defined its pro-proliferative effects. A study investigating the influence of ET-1/ETAR on YAP/TAZ in colorectal cancer described results exhibiting this attribute [21]. Yes-associated protein (YAP) is an oncoprotein, associated with poor prognosis and signals downstream of the Hippo pathway, and TAZ is a transcriptional coactivator with a PDZ-binding motif. The Hippo pathway is important in regulating cell proliferation, migration and apoptosis [20,21,22]. In normal cellular signalling of the Hippo pathway, LATS1/2 kinases would phosphorylate YAP and TAZ, restricting their localisation to the cytoplasm. This study found that ET-1 signalling through ETAR, coupled to Gαq/11, inhibited the LATS1/2 kinases, resulting in the dephosphorylation of YAP/TAZ, permitting the nuclear accumulation of this axis (Figure 3) [21]. The localisation of YAP/TAZ in the nucleus results in their binding to the transcription factor family TEA domain (TEAD), which induces the gene expression of connective tissue growth factor (CTGF) and cysteine-rich angiogenic inducer 61 (CYR61), both belonging to the CCN protein family and regulating cell proliferation, migration and angiogenesis [21,23]. The authors suggested the association of ET-1 with YAP/TAZ was cell-type-specific, with these results exhibited in the human colorectal carcinoma cell line HCT116 in vitro and in vivo in xenograft nude mice models [21]. Supporting results were also presented in a review of high-grade serous ovarian carcinoma (HG-SOC) cells [17]. Contrastingly, these results were not exhibited amongst breast and prostate cancer cell lines, and it was proposed the reason for this was ETAR was not overexpressed to the extent it was in the colorectal carcinoma cell line, thus not displaying the same action [21].

ET-1 has been shown to promote the metastatic phenotype in multiple cancers, such as ovarian, breast, bladder, bone, colon and liver [24,25,26,27,28,29,30,31]. A study defined the physical interaction between the transcription factor zinc-finger E-box-binding homeobox 1 (ZEB1) and YAP induced by ET-1 (Figure 3). They revealed the ability of ET-1 to coordinate the localisation of YAP/ZEB1/TEAD4 to JUN, which composes part of the AP-1 transcription factor. AP-1 has been shown to promote the expression of *EDN1*, highlighting a positive feedback mechanism of this signalling pathway [26]. Another component, integrin-linked kinase (ILK), is upregulated downstream of ET-1 signalling and is shown to facilitate the nuclear localisation of ZEB1 through the ET-1 axis. Transwell cell invasion assays concluded ET-1 promoted metastatic behaviour by inducing the epithelial–mesenchymal transition (EMT) in HG-SOC in vitro and in vivo through the ZEB1/YAP/AP-1/ILK axis [26].

Furthermore, the ET-1 axis has been implicated in the induction of EMT, affecting transcription factors such as Snail and ZEB1, which are key to promoting EMT, switching the cellular phenotype to acquire mesenchymal traits [26]. A positive correlation has been discovered between ETAR and ZEB1 expression in HG-SOC patients, and the regulatory role of miR-200b/c in this expression has been defined [32]. The regulation of ETAR expression by ZEB1 was enacted through ZEB1’s inhibition of miR-200b/c. The authors concluded there to be an axis composed of ETAR–ZEB1–miR-200b/c induced by ET-1, which mediates cellular plasticity and extracellular matrix (ECM) degradation by matrix metalloproteinases (MMPs). The upregulation of ET-1 has been shown to increase ZEB1 mRNA and protein expression in the HEY ovarian cancer cell line. ET-1-induced regulation of ZEB1 expression was blocked upon treatment with ET-1 receptor antagonists targeting ETAR [32]. Studies reported an increased expression of mesenchymal markers such as vimentin and N-cadherin alongside E-cadherin repression through ET-1 action, but this was inhibited upon macitentan (a dual ET-1 receptor antagonist) addition (Figure 3) [32]. In vivo studies using ovarian cancer mice models found a metastatic impairment when they were treated with macitentan, highlighting the potential to target and prevent the action of this ligand through repurposing this drug for cancer therapy [32].

ET-1 signalling through the GPCR ETAR, coupled to Gαq/11, activates the GTPase Rho, which inhibits the kinase activity of LATS1/2 through the Hippo pathway [21]. Rho coordinates actin cytoskeletal remodelling and plays a role in the degradation of the basement membrane to produce invadopodia. These protrusions encourage the invasive behaviour of tumour cells and are mediated by ETAR/β-arr1 [17,25]. A study using RNA interference (RNAi) in the A549 lung cancer cell line to silence ET-1 found a decrease in the protein expression of RhoA/C [9]. This is consistent with the data obtained by Semprucci et al. (2016), in which ET-1 stimulation amplified the RhoA/C expression, which was prevented by the addition of macitentan in the epithelial ovarian cancer (EOC) cell line HEY [25]. The authors determined RhoC had the most prevailing effect on invadopodia maturation, whereby silencing this enzyme inhibited ET-1-induced invadopodia development, consequently negatively impacting the invasive capacity of these cells. Further approaches led to the characterisation of the mechanism by which ET-1/β-arr1 promotes metastatic behaviour. ET-1/ETAR associates with β-arr1, PDZ-RhoGEFs and RhoC to stimulate Rho-associated coiled-coil-forming kinase (ROCK) and LIM kinase (LIMK), which promote cofilin phosphorylation (Figure 3) [25]. The role of cofilin is to depolymerise actin filaments in its active state, but upon LIMK action, cofilin is inactivated, permitting actin polymerisation and promoting invadopodia formation [25,33]. The colocalization of the actin regulators membrane-type 1 MMP (MT1-MMP), cortactin and Tks5, which are recruited to the F-actin was observed upon ET-1 signalling. The upregulation of protease activity is also exhibited downstream of ET-1 to promote ECM degradation through MMP-2 and -9 [25]. In EOC mice xenografts, treatment with macitentan caused a 90% decrease in tumour growth over a 56-day window, where metastatic lesions were decreased in comparison to in the controls. It was suggested the impairment of metastatic behaviour was linked to cofilin phosphorylation, as this was inhibited. Metastases were significantly depleted in EOC xenografts subject to dual therapy of macitentan and a ROCK inhibitor, highlighting the opportunity to use this as a therapeutic target in clinic [25]. The results from this study highlight the potential anticancer role of macitentan within EOC to inhibit the metastatic phenotype. Thus, this may represent one of the ways in which ET-1 can be targeted to improve cancer treatments, and further research on this in a clinical setting would be intriguing.

Moreover, ILK is a critical player in invadopodia formation and has a guanine nucleotide exchange factor (GEF) partner, βPIX, and is regulated downstream of ET-1/β-arr1 through ETAR. Applications of ET-1 receptor antagonists confirmed the specific GPCR responsible for this association; ambrisentan (an ETAR antagonist) prevented this observation, whereas BQ-788 (an ETBR antagonist) permitted this complex formation [24]. It was found the tyrosine kinase Src complexes with β-arr1 and targets βPIX, activating it, where this subsequently activates Rac3 (GTPase). ILK/βPIX/Rac3 acts to phosphorylate and activate PAK1, leading to cofilin phosphorylation and successive actin polymerisation, as shown in Figure 3. Interestingly, ET-1 induces cortactin localisation to F-actin regions in SCOV3 and OVCAR3, supporting previous efforts showing this in other ovarian cancer cell lines [24,25]. MT1-MMP was a confirmed substrate of ILK and is regulated by ET-1, as the phosphorylation of MT1-MMP was absent after ambrisentan treatment. According to a series of silencing reactions and ambrisentan treatment, the migrative and invasive ability of SKOV3 cells across the mesothelium was monitored, and it was concluded ET-1 signalling induced greater migration through the ILK/βPIX/Rac3 axis. In serous ovarian cancer (SOC) mice xenografts utilising the SKOV3 cell line, the abundance of metastatic lesions exhibited in the abdomen was significantly higher in controls than in SKOV3 cells treated prior with ambrisentan [24]. In targeting ET-1, the metastatic phenotype in different ovarian cancers is impaired, hindering the invasive capacity. Repurposing of ambrisentan, an approved pulmonary hypertension drug, could present an avenue for utilising the positive anticancer effects ET-1 can have when inhibited [24]. This underlines the significance of further research and exploring how ambrisentan would perform in clinical trials from an oncology stance.

### 3.2. Angiogenesis

For cancer metastasis to be successful, establishing a new vascular network is vital for tumour cell survival. Angiogenesis is the term used to describe the process of new blood microvessel formation from existing ones [34]. Tumour-cell-secreted factors stimulate the angiogenic pathway to promote the rapid growth and formation of new microvessels in a state of uncontrolled cell proliferation [34]. ET-1 is a factor which has been reported to be overexpressed by tumours [35]. By means of autocrine and paracrine signalling, ET-1 can promote tumour angiogenesis directly and indirectly, stimulating both early and late angiogenic processes [9,17]. The angiogenic pathway is often stimulated under hypoxic cellular conditions via the activation of the heterodimer hypoxia-inducible factor-1 (HIF-1) [36]. Multiple studies have demonstrated ET-1 can emulate an environment comparable to hypoxia and stimulate the release of VEGF equipotently to HIF-1α [20,37]. Under normoxia, hydroxylation by the HIF prolyl hydroxylase domain 2 (PHD2) enzyme continually degrades HIF-1α [7,38]. In hypoxic conditions, hydroxylation does not occur, permitting HIF-1α’s stability. ET-1 stabilises HIF-1α expression through the inhibition of PHD2 in normoxia and hypoxia, promoting angiogenic-stimulating conditions [7,38]. Subsequently, HIF-1α promotes the expression of vascular endothelial growth factor (VEGF) through interaction with its promoter [36]. VEGF is a potent mitogen and a pivotal angiogenic growth factor; thus, HIF-1α activation is an important aspect of this tumour progression pathway [36,37].

An in vitro study involving the OVCA 433 cell line (human ovarian carcinoma cell) found the stimulation of VEGF production using ET-1 increased it twofold compared to in the controls. The authors also determined VEGF stimulation by ET-1 must signal through ETAR, as upon the addition of the ETAR antagonist BQ-123, VEGF production was inhibited [37]. Similar efforts were shown in an earlier study, which concluded that mitogenic action must signal through ETAR, determined in a similar experiment harnessing BQ-123 [39]. The results of a further study investigating lung cancer were in agreement with the conclusion that ET-1 stimulates VEGF release. It was shown by silencing ET-1 using RNAi that the VEGF expression decreased, and consequently the cell proliferation of A549 cells was impaired [9]. The same results were demonstrated in an in vivo model using two groups of BALB/c nude male mice; one group received a transfection of A549 cells and a second a transfection of ET-1-silenced A549 cells. The tumours with silenced ET-1 proliferated at a significantly slower rate (*p* < 0.05) [9]. A further study discovered that ET-1 promoted tyrosine phosphorylation on vascular endothelial growth factor receptor 2 (VEGFR2), but this was inhibited upon the addition of macitentan or the knockout of β-arr1 [19].

In another recent study focusing on HG-SOC, ET-1 was hypothesised to affect the nuclear localisation of the YAP/p53/HIF-1α complex [20]. In PMOV10 cells carrying TP53 mutations, YAP and mutp53 form transcriptional machinery through the nuclear linkage constructed by β-arr1, which have been shown to be transcriptionally co-dependent, as driven by the ET-1 axis [20]. The authors demonstrated an interdependency between HIF-1α and YAP nuclear localisation, promoted by ET-1 signalling in HG-SOC cells through interference with large tumour suppressor kinase 1 (LATS1); silencing HIF-1α or YAP inhibited the accumulation of YAP and HIF-1α, respectively, in the nucleus [20]. It was concluded that YAP stabilised the structure of the HIF-1α protein, reinforcing the notion of dependency between YAP and HIF-1α. The downregulation of HIF-1α expression was observed upon YAP reduction, thus reducing VEGF expression, a main downstream target of HIF-1α signalling. The nuclear localisation of these is crucial for transcriptional reprogramming, altering the phenotype of the cell to enrich angiogenic processes [20]. To elucidate this relationship further, in vivo testing would be beneficial, as well as harnessing other models of angiogenesis to verify this mechanism. Characterising how ET-1 affects this axis within in vivo models may aid in the development of therapeutic targets. Novel therapeutic targets are vital, as cancer drug resistance is a prevailing issue. ET-1 has been implicated in promoting drug resistance, and thus further exploration of how this peptide manifests this phenotype may aid in the development of novel targets which can be exploited.

### 3.3. ET-1 in Drug Resistance

The interaction of ET-1 with the signal inducer β-arr1 has been suggested to harbour a route for drug escape, promoting chemoresistance and apoptosis evasion [19]. Rosano et al. (2014) set out to characterise the role of the ET-1 axis in facilitating the chemoresistant phenotype exhibited in many types of cancer in the context of β-arr1. Epithelial ovarian cancer (EOC) cell lines A2780 and OV2008 exhibiting resistance to cisplatinum and paclitaxel (taxol drug) expressed increased levels of ETAR and β-arr1 compared to those with a sensitive phenotype [19]. Sensitive and resistant cells were treated with ET-1, and an increase in β-arr1 and β-catenin nuclear localisation was observed in both [19]. Transcriptional machinery is activated upon β-catenin nuclear accumulation, associating with the TCF/LEF transcription factors, but this was prevented upon β-arr1 silencing or macitentan treatment. Action through ETAR is evident as, upon treatment with BQ-788, an ETBR antagonist, this activity was still permitted [19]. This suggests that the overexpression of ETAR could be a predictive marker of resistance to chemotherapeutics [17,19]. ET-1 was also shown to promote the stabilisation of β-catenin in gallbladder cancer (GBC) cell lines, but this was inhibited with the addition of macitentan [40]. Furthermore, combination therapy harnessing macitentan and cisplatinum brought about higher levels of the apoptotic markers cleaved poly ADP-ribose polymerase (cl-PARP) and cleaved caspase-3 (cl-caspase 3). These results highlight the potential use of macitentan in cancer therapeutics and how it may be used to improve the response to other anticancer drugs. Moreover, upon this cotherapy, Bcl-xL (an anti-apoptotic protein) expression saw a decrease upon ET-1 blockade. This indicates ET-1 induces apoptosis evasion upon chemotherapeutics, fostering drug resistance through the ET-1/β-arr1 complex [19]. Additionally, β-arr1, along with the activity of Rho, which reorganises the actin cytoskeleton, facilitates the nuclear accumulation of YAP/TAZ/TEAD downstream of ET-1, which form pro-survival transcriptional machinery to amplify the expression of genes which evade chemotherapeutics [17].

The ET-1 axis is activated in pancreatic adenocarcinoma, which is an aggressive form of cancer and is often difficult to treat, as diagnosis often takes place in the late stage of the disease due to the lack of symptoms exhibited in patients with this disease. Gemcitabine (chemotherapy drug) is often the first-line treatment, but resistance is quickly acquired. A study investigated the effects of targeting the ET-1 axis within this disease [41]. Upon a dose-dependent increase in gemcitabine, the transcriptional activity of *EDN1* and EDNRA was upregulated with each increased dose, accompanied by the enhanced expression of CTGF and CYR61. The expression of EDNBR remained unchanged upon the dose-dependent increase in this chemotherapeutic agent [41]. It is thought that the ET-1 axis sustains chemoresistance through facilitating anti-apoptosis mechanisms, promoting EMT and the invasive capacity of the cells. The application of combination therapy in AsPC-1 cells using gemcitabine and bosentan (a dual ET-1 receptor antagonist) saw an increase in cl-PARP, indicating the increased activity of apoptosis [41].

Moreover, ET-1 was shown to promote drug resistance in the lung cancer cell line, DMS114. DMS114 cells with resistance to nintedanib (a kinase inhibitor used to block cancer growth) (DMS114/NIN) portrayed an amplified expression of *EDN1* and *EDNRA*, encoding ET-1 and ETAR, respectively. DMS114/NIN cells exhibited the hyperphosphorylation of PKC/NF-κB, thus promoting p65 expression downstream of ET-1 action, resulting in protein expression of ATP-binding cassette transporter B1 (ABCB1) [42]. ABCB1 actively permits the efflux of the small molecule nintedanib, thus composing the machinery responsible for nintedanib resistance in DMS114 cells. Treatment with the dual ET-1 receptor antagonist tezosentan blocked this phosphorylating action of ET-1 and further downregulated the expression of ABCB1, resulting in the re-sensitisation of DMS114 cells to nintedanib [42]. This study highlights the opportunity to repurpose tezosentan to target ET-1 in cancer. This drug may have the potential to tackle drug resistance, which is a critical issue in cancer therapy.

Another recent study outlined the significance of ET-1 signalling within GBC for the first time. The expression of both ETAR and ETBR was found abundantly, but it was concluded ETAR’s contribution to GBC progression was more predominant and correlated with a poorer prognosis [40]. Th expression of EMT markers such as Snail, Slug and ZEB1 was upregulated, with E-cadherin repression observed alongside this as mediated by ET-1 signalling (Figure 3). The MMP-9 transcription levels were repressed upon macitentan treatment, indicating ET-1 signalling regulates the expression of this enzyme. ET-1 contributes to the invasiveness of GBC cells, and blockade of its receptors with macitentan was shown to inhibit this trait, but only in cells which had manifested the invasive phenotype [40]. The induction of EMT is associated with enhanced chemoresistance. Upon gemcitabine treatment, ETAR and ETBR expression was enhanced, alongside that of key ET-1 signalling markers such as ZEB1, NF-κB and β-catenin. Combination therapy of macitentan and gemcitabine blocked the upregulation of these components and reduced the cell viability of GBC cells to a greater extent. It was ultimately suggested ET-1 contributes to the resistance of chemotherapeutics often exhibited in GBC and the blockade of ET-1 receptors permits sensitisation to this drug class [40].

ET-1 is involved in the promotion of drug resistance across many human cancers, and blockade of its signalling has shown promise in vitro in improving the response to other anticancer drugs such as chemotherapeutics. This highlights ET-1 as a promising therapeutic target which could provide a new strategy for cancers which no longer respond to conventional treatments.

## 4. Therapeutic Approaches

There are several inhibitors approved for targeting ET-1 signalling in the context of other diseases, like pulmonary arterial hypertension. There are indications from in vitro and in vivo studies that ET-1 receptor antagonists have shown promise in targeting this peptide from an oncological perspective. Several inhibitors have been tested in clinical trials; however, there has been no success as of yet in showing the efficacy of these drugs in human cancers.

### 4.1. Small Molecule Inhibitors and Drug Repurposing

Drug repurposing is an attractive construct for the pharmaceutical industry in more ways than one. By giving new uses to drugs that are already approved and safe for administration, this eliminates many limitations and challenges surrounding drug discovery, such as the duration it takes to design new molecules and the cost of this process, as well as the toxicity issues which often arise in clinical trials [43]. ET-1 receptor antagonists may have the potential to improve the response to other anticancer drugs such as PARP inhibitors (PARPi) and chemotherapy drugs in cases where cells exhibit chemoresistance [17,19,20].

In vitro treatment of the HG-SOC cell line PMOV10, a dual receptor antagonist targeting both ETAR and ETBR, macitentan, a drug approved for treating pulmonary arterial hypertension, was shown to inhibit the gene expression of VEGF, ET-1 and CTGF through the YAP/HIF-1α/mutp53/β-arr1 network by preventing the accumulation of HIF-1α and YAP in the nucleus [17,20]. Consequently, the metastatic properties of the HG-SOC cells were significantly reduced by the addition of macitentan, which blocked ET-1 signalling [20]. The action of macitentan also improved the response of the HG-SOC cells to chemotherapy by interfering with YAP/TAZ (Figure 3) [17]. Moreover, PMOV10 cells treated with macitentan exhibited an increased sensitisation to the PARPi drug Olaparib, confirmed by increased levels of the apoptotic markers cl-caspase 3 and cl-PARP. This suggests ET-1 provides a DNA damage escape pathway for cancer cells to evade apoptosis. This observation was consistent in the MDA-MB-468 breast cancer cell line, as well as in HG-SOC patient-derived xenografts (PDX) and OVCAR3 xenografts, where the size and number of tumour nodules decreased in vivo [20]. Furthermore, it has been alluded to that macitentan can interfere with the β-arr1 signalling network by blocking the ET-1 receptors, preventing ET-1 signalling and improving the response of cancer cells to platinum-based chemotherapy, as well as downregulating angiogenesis and metastatic action in different cancer types [17,18].

A study investigated the effects of combination therapy utilising the type-2-diabetes-approved drug metformin (MET) and simvastatin (SVA), which is a statin used to control cholesterol levels and treat CVD. These two drugs have both exhibited anticancer properties [38]. MET has been shown to exert an anti-proliferative effect on tumours, promote normoxia and induce apoptosis. Moreover, statins have exhibited anticancer properties in colorectal, breast and human melanoma cancer through prevention, apoptosis induction and impairing the metastatic ability, respectively [38]. Investigating the cell lines murine mammary carcinoma 4T1 and murine melanoma B16, HIF-1α expression was inhibited and a significant decrease in hypoxia was exhibited upon MET and SVA treatment. This treatment was shown to repress the expression of *EDN1* and found a reduction in ET-1 and ETBR. Additional treatment in the HepG2 liver cancer cell line and MCF7 breast cancer cells established the combination of MET and SVA interfered with the ET-1-induced nuclear localisation of HIF-1α, along with PHD2 upregulation, resulting in increased degradation of HIF-1α, reducing angiogenesis [38]. In vivo combination treatment in female BALB/cAnNCrl and C57BL/6J mice showed a decrease in tumour cell proliferation. This anti-growth effect was also exerted on lung, liver and cervical cancer cell lines. Increased apoptotic activity was further exhibited in multiple cancer cell lines downstream of the combination treatment through interference with ET-1, supported by repressed survivin expression and increased levels of cl-caspase 3 [38]. ET-1-induced effects were inhibited by the combination treatment to an extent comparable to the ET-1 dual receptor antagonist bosentan in regard to HIF-1α, survivin and ETBR expression but greater than bosentan in relation to cell proliferation [38]. The same effect of the combination treatment was exhibited in breast cancer patient-derived organoids (PDOs), and it was suggested the mechanism for this was through preventing ET-1’s induction of HIF-1α expression, thus preventing the angiogenic pathway via the ET-1/ETBR axis [38].

To date, there has been a collection of clinical trials which have been carried out using ET-1 receptor antagonists, targeting ETAR and ETBR. Table 1 composes a summary of the trials carried out targeting ET-1 signalling (Table 1). There are currently two clinical trials ongoing, with the first targeting ETBR-positive tumours, which contribute to immunomodulatory therapy resistance by inhibiting T-cell extravasation and tumour infiltration [44]. A second ongoing phase 1 study is monitoring the addition of the dual ET-1 receptor antagonist bosentan in combination with two chemotherapy drugs. The idea is to improve the response to chemotherapeutics in unresectable pancreatic cancer by blocking ETAR/ETBR (NCT04158635).

A trial which terminated in 2015 researching advanced biliary tract cancer (ABTC) had an interesting approach of using SPI-1620, an analogue of ET-1 which is highly selective to ETBR, in combination with docetaxel (NCT01773785). It had been suggested in prostate and breast tumour models that this agonistic action against ETBR within the tumour vasculature increases blood flow, improving the drug delivery of anticancer agents [45,46]. This trial unfortunately did not produce any significant results, with toxicity problems experienced [47].

### 4.2. Polyphenols as Antitumour Agents

Cancer therapies such as chemotherapy present various challenges, including severe side effects, resistance to chemotherapeutic drugs impacting their efficacy and impaired tumour clearance. Subsequently, continued efforts to improve techniques such as chemotherapy are ongoing [48]. One such effort is to supplement chemotherapy with polyphenols as anti-tumour agents. Polyphenols are phytochemicals with complex structures composed of catechol and gallol groups, possessing multiple hydroxyls, giving rise to their potent antioxidant properties. Their structures allow the formation of many different interactions, allowing them to bind to various substrates [48].

A recent study harnessed combination therapy of curcumin (CUR), a polyphenol, and cisplatin (CIS), a chemotherapy drug. In vivo studies using DMBA to induce ovarian cancer in rat models were examined subsequent to tumour formation [49]. The ET-1 mRNA levels were enriched within these tumours, but upon cotherapy of CUR and CIS, this was repressed. Interestingly, ETBR mRNA expression was significantly upregulated by this combination treatment, but this observation was not consistent for ETAR mRNA expression [49]. Nuclear-factor-erythroid-2-related factor (NRF2) mRNA expression was further upregulated in an in vitro model using SKOV3 cells upon CUR and CIS treatment but not in the cells treated with CIS alone. The authors concluded CUR induced NRF2 expression, which mediated the upregulation of ETBR expression, promoting ET-1 clearance. In vivo models exhibited a reduction in epithelial markers including E-cadherin and β-catenin, but upon CUR and CIS treatment, their expression was restored, and the N-cadherin (a mesenchymal marker) expression was diminished [49].

This highlights a potential therapeutic avenue for targeting ET-1 expression; however, the delivery of CUR requires further assessment, as this substance has low bioavailability. This may be overcome by harnessing nanoparticles as carrier molecules [49]. Moreover, CUR possesses other therapeutic benefits, as it may act as a supplement to chemotherapy administration. CUR has exhibited renal protective attributes, which may aid in mitigating the injuries often inflicted upon the kidneys during this harsh anticancer treatment, indicating the multiple potential benefits this compound could have if utilised [49].

An earlier study investigated the antitumour activity of the green tea polyphenol epigallocatechin-3-gallate (EGCG) in ovarian carcinoma in targeting ETAR/ET-1 [50]. ET-1 has previously been shown to promote angiogenesis and invasion by activating the cyclooxygenase (COX)-1 and COX-2 enzymes, which produce prostaglandin (PG) E2 and VEGF downstream of their activation [50]. In vitro studies of EGCG treatment on the HEY and OVCA 433 ovarian carcinoma cell lines exhibited a dose-dependent reduction in the ET-1 and ETAR mRNA and protein levels. In addition to this, treatment with EGCG on the HEY cell lines found a dose-dependent reduction in the ET-1-induced COX-1/2 expression levels. COX-1/2’s enzymatic action was also inhibited, as a similar reduction was seen in the ET-1-induced PGE2 levels [50]. The reduction in PGE2 levels was equivalent to that inflicted by the selective ETAR receptor antagonist BQ-123. Thus, the authors concluded this polyphenol could hinder ETAR-mediated PGE2 production through COX-2 in ovarian carcinoma cells. This highlights the potential therapeutic relevance of polyphenols such as green tea EGCG and its anticancer attributes it can inflict by targeting the ET-1 axis [50].

Thus, polyphenols such as CUR and EGCG represent a potential avenue for targeting ET-1 as antitumour agents. This creates a basis to expand upon this research in terms of whether other polyphenols retain the ability to target ET-1 both in vitro and in vivo. Exploring the anticancer properties of EGCG, as well as the combined action of CUR with chemotherapy agents, in clinical trials to target ET-1 would be interesting. Investigating the potential benefits these compounds may offer could provide valuable insight into their therapeutic significance.

### 4.3. Gene Therapy

Epigenetic regulation can mediate key phenotypical changes within cells, often through transcriptional regulation, with the ability to promote tumorigenesis. microRNA (miRNA/miR) are one type of epigenetic modulator, regulating gene expression at the post-transcriptional level. They are short non-coding RNA molecules which target the 3′-untranslated region (UTR) of mRNA, where they bind to complementary sequences and mediate mRNA degradation or the repression of translation [51]. A single miRNA can regulate numerous genes, and a single gene can be regulated by multiple miRNAs. Classifying an miRNA as a tumour suppressor or an oncogene depends on the target gene it influences [52]. miRNAs as therapeutic targets are emerging and are a key focal point of the current research. In targeting ET-1, these hold many advantages over treatments harnessing ET-1 receptor antagonists, as miRNA intend to restore physiological function rather than blocking a receptor, resulting in fewer side effects and lowering the treatment toxicity. Furthermore, miRNA is suggested to have more powerful, persisting effects, as these molecules are more stable than ET-1 receptor antagonists [53].

Multiple miRNAs which regulate the *EDN1* gene were found to be downregulated in endometrial cancer, significantly miR-130a-3, which has been said to have anticancer abilities [54]. Without this miRNA control, *EDN1* overproduction is permitted, and this was consistent for EDNRA but not for EDNRB, as its expression was repressed, assessed using mRNA microarray and validated using RT-qPCR [54]. Furthermore, miR-1 can act as a tumour suppressor and was seen to target and regulate ET-1 expression in hepatocellular carcinoma cell lines when transfecting HepG2 and HepG3 with overexpressed miR-1 [53]. Upon this approach, the ET-1 precursor preproET-1 saw a reduction in protein expression in both cell lines. Proliferation in the HepG2 and HepG3 cell lines was inhibited downstream of miR-1 overexpression, concluding miR-1 regulation of ET-1 can control proliferation in hepatoma cells [53].

The role of miR-1 in regulating ET-1 in nasopharyngeal carcinoma (NPC) was later defined. A catalytic component of the Polycomb repressive complex 2 (PRC2) known as Enhancer of Zeste Homolog 2 (EZH2) was of interest in this study [55]. PCR2 methylates histone H3 on lysine 27, which induces gene silencing, and EZH2 retains this histone methyltransferase activity. High expression of EZH2 has been associated with many solid tumours and predicts a worse prognosis and more aggressive phenotype. This study confirmed EZH2 was expressed at much higher levels in NPC cell lines compared to non-cancer cell lines [55]. qRT-PCR profiling studies revealed miR-1 to have low expression in 6-10B NPC cells expressing high levels of EZH2. In the 5-8F NPC cell line with silenced EZH2, miR-1 was abundantly expressed, revealing an inverse relationship between EZH2 and miR-1 expression. Using reporter and chromatin immunoprecipitation assays, it was determined that EZH2 could directly interact with the promoter of miR-1 and negatively regulate its expression [55]. The authors of this study investigated miR-1 regulation in relation to ET-1 in NPC, based upon previous findings by Li et al. (2012) [53]. The respective study found ET-1 to be significantly upregulated in a grade-specific manner across NPC tissues. They found the ET-1 protein levels were significantly downregulated upon the ectopic expression of miR-1 [55]. Furthermore, a positive relationship was determined between EZH2 and ET-1 expression using ELISA and Western blot analyses. Upon EZH2 overexpression in NPC cells, ET-1 also had enhanced expression, and the knockdown of EZH2 coincided with a decrease in ET-1 protein levels [55]. It was concluded that EZH2 induced angiogenesis by mediating ET-1, and tumours with higher ET-1 expression presented with a greater microvessel density (MVD). The mechanism by which this regulation occurs remained undefined within this study but presented a novel pathway through which EZH2 induces angiogenesis, by inhibiting the miR-1/ET-1 axis [55]. This presents the opportunity for novel therapeutic targets and for further studies to characterise this molecular mechanism for a greater understanding of angiogenesis in NPC.

Using Western blot analysis subsequent to miR-19b-3p mimic transfection in the gastric cancer (GC) cell line SGC-7901, a further study evidenced ETBR expression is significantly downregulated upon this miRNA’s overexpression. Interestingly, VEGF-A expression coincided with ETBR downregulation upon miR-19b-3p upregulation. These results indicate miR-19b-3p may function as an oncosuppressor by negatively regulating ETBR expression, consequently reducing VEGF-A and modulating angiogenesis in GC [56]. In another recent study involving GC, miR-648 was found to be downregulated in the tissues of this cancer, reversely correlated with ET-1 upregulation. Following chemotherapeutic intervention using 5-fluorouracil (5-FU), miR-648 was upregulated, paired by a reduction in ET-1 levels. In the MKN-45 cell line exhibiting chemoresistance, 5-FU inflicted little effect on ET-1 expression. However, upon the combined transfection of miR-648 with 5-FU, ET-1 saw a significant downregulation in the context of mRNA and protein expression. These results suggest the enrichment of the cells with miR-648 restored sensitivity to the chemotherapeutic 5-FU and inhibited proliferation [51].

Moreover, miR-30a has been defined as inhibiting chemoresistance in ovarian carcinoma cells through ETAR regulation, as well as cellular plasticity and invasion. The OV2008 CIS and A2780 CIS cell lines exhibiting resistance to cisplatinum (CIS) were used to explore miR-30a regulation [57]. ETAR was identified as a direct target of miR-30a, and an inverse correlation was observed in terms of ETAR and miR-30a expression. OV2008 CIS and A2780 CIS expressed low levels of miR-30a, simultaneously with high levels of ETAR [57]. Upon miR-30a inhibition of ETAR, the ET-1-induced phosphorylation of p42/p44 MAPK and Akt was reduced in the A2780 and A2780 CIS cells. When phosphorylated and activated, these components signal to promote survival and apoptotic protection [57]. In A2780-sensitive and A2780-resistant mutants, the increased expression of miR-30a coincided with the abundance of apoptotic markers such as increased cl-PARP and cl-caspase 7. This observation was enhanced upon the addition of CIS, suggesting miR-30a can re-sensitise cells to chemotherapy treatments by targeting ETAR [57]. Another effect miR-30a overexpression brought about was reversing EMT. The e-cadherin levels increased in the OV2008 and A2780 CIS-resistant cells, alongside the repression of Snail and vimentin expression [57]. ET-1-induced VEGF expression was also prevented upon the overexpression of this specific miRNA. The authors further demonstrated miR-30a overexpression prevented the production of structures resembling vasculature downstream of ET-1/ETAR [57]. In in vivo studies using OV2008 CIS cells transfected with miR-30a and a control cell line, these were injected into nude mice. The group with overexpressed miR-30a presented with a significant decrease in tumour weight and growth compared to the control group, as well as decreased ETAR expression and MAPK and Akt activation [57]. Another study highlighted the potential ability to target ETAR expression through the use of luciferase reporter assays with ovarian cancer cells and the application of miR-200b/c, which caused the downregulation of ETAR expression [32]. This indicates there is an important level of regulation here and that it would be interesting as a therapeutic intervention. These data highlight the potential to use different miRNAs to target the ET-1/ETAR axis to improve response to cancer therapeutics and reduce the aggressiveness of ovarian carcinoma.

The results of many of these studies highlight the multiple ways in which different miRNAs can regulate ET-1 expression to promote oncogenesis. Regulating the ET-1 expression using miRNA could be approached by targeting *EDN1*, as well as targeting ET-1 receptor expression, such as through EDNRA. This draws interest in exploiting them for the regulation of ET-1 signalling from a therapeutic stance. Many preclinical studies have shown this to have positive therapeutic benefits. Therefore, further studies using in vivo models, larger patient cohorts and differing cancer types would be beneficial to evaluate the significance of these as a cancer therapeutic option.

## 5. Future Directions

### 5.1. ET-1 in Diagnosis

Within systemic circulation, ET-1 has a very short half-life, limiting the potential to use ET-1 as a diagnostic factor [58]. One study suggested big-ET-1 (a precursor of ET-1) had a much longer half-life than ET-1, making it more stable in circulation and proposing the role of it as a biomarker for early breast cancer diagnosis [58]. Contrary to this, studies have shown big-ET-1 to have a poorer circulatory half-life than ET-1 but slower tissue clearance within the renal and hepatic system (reviewed in [11]).

The characterisation of proET-1 peptides for use as biomarkers of ET-1 synthesis was performed by Yuzugulen et al. (2017) [59]. *EDN1* gene expression as induced by TNF-α resulted in the upregulation of the proET-1 peptides N-terminal-proET-1 (NT-proET-1) and C-terminal-proET-1 (CT-proET-1) but had no effect on endothelin-like domain peptide (ELDP) and ET-1. These peptide fragments are depicted in Figure 1. However, a correlation was found between the ELDP and CT-proET-1 plasma concentration, which was consistent with ET-1 synthesis [59]. ELDP and CT-proET-1 are more stable in circulation than other proET-1 peptides and exhibit two phases of clearance [59]. Phase 1 has estimated half-lives of 0.5 and 0.7 min and phase 2 half-lives of 5.7 and 7.3 min for ELDP and CT-proET-1, respectively. These ET-1 precursors have a much slower clearance rate compared to ET-1, with a short half-life of 1 min [11,59]. ELDP has been suggested to enhance ET-1 signalling and hinder its therapeutic targeting by promoting resistance to endothelin receptor antagonists [59]. It has been suggested that ELDP may possess the most resemblance to ET-1 and therefore represent a better biomarker for ET-1 expression [11]. CT-proET-1 has been defined as a prognostic factor in chronic heart failure patients, thus generating interest as to whether this can additionally be used as a biomarker in cancer model. These authors demonstrated CT-proET-1 was a superior biomarker to ELDP and exhibited increased sensitivity, with better diagnostic potential in categorising heart failure severity as well as inflammatory extent [59]. Therefore, further research exploring these two peptides as biomarkers of ET-1 signalling would be interesting from an oncological stance.

Conversely, Irfan et al. (2023) were able to distinguish differentiating grades of oral squamous cell carcinoma (OSCC) according to salivary ET-1 levels. An incremental trend in the ET-1 levels was exhibited in groups with increased malignancy of OSCC [60]. Nevertheless, since the synthesis of ELDP and CT-proET-1 is correlated with ET-1, it would be interesting to examine their levels as potential diagnostic factors in various cancers.

### 5.2. Extracellular Vesicles (EVs) to Target ET-1

Mesenchymal stromal/stem cells (MSCs) secrete extracellular vesicles (EVs) and have been a key point of interest in the research in terms of whether these can be exploited for more targeted therapy. MSC-derived EVs are vehicles for many different cargo molecules, such as RNA, lipids and proteins, and are involved in cell–cell communication [61]. MSCs have low immunogenicity, which makes them attractive drug carriers for enhancement and more targeted delivery within a patient. The vesicles can be enriched with anticancer drugs such as chemotherapeutic agents for more targeted cancer therapy [61,62]. Preclinical studies have shown a positive therapeutic response in targeting ET-1 expression by harnessing EVs. Thus, this may pose an attractive strategy for targeting ET-1 signalling in cancer.

Through the enrichment of MSC-derived EVs with specific miRNA, Vieira et al. (2022) demonstrated the upregulation of angiogenic proteins such as ET-1, PGF and artemin [63]. Lentiviral vectors of miR-210 caused their overexpression in MSC-derived EVs. Subsequent techniques involving the application of these EVs to human umbilical vein endothelial cells (HUVECs) in a membrane-based sandwich assay resulted in ET-1 upregulation, along with the upregulation of other angiogenic proteins [63]. This is an interesting avenue through which to target ET-1 expression and poses the question of whether this strategy could be used to enrich EVs with alternative miRNA to regulate the repression of ET-1, preventing the upregulation of many cancer-promoting pathways that ET-1 is involved in.

A further study examined the miRNA profile across exosomes (vesicles with a 150 nm or smaller diameter) secreted from the ovarian cancer cell lines SKOV3 and A2780 compared to the immortalised ovarian epithelial cell line IOSE-80 [64]. The miR-92b-3p expression was significantly downregulated in the exosomes derived from the cancerous cell lines (SKOV3/exo and A2780/exo) compared to the epithelial cell line exosomes, ISOE-80/exo. The SKOV3 exosomes were manufactured with enhanced miR-92b-3p expression (SKOV3-92B/exo), and HUVECs were subjected to them. This prevented the HUVECs from undergoing migration and angiogenesis, shown using a migration assay and a tube formation assay [64]. The authors concluded that one way in which ovarian cancer cells stimulate angiogenesis is through the delivery of exosomes with a lower expression of miR-92b-3p [64]. A direct target of miR-92b-3p is the transcription factor SOX4, which belongs to the Sry-related high-mobility group box (SOX) family. SOX4 has been shown to be directly associated with the ET-1 promoter and induce its activity. In addition, it has been shown to induce the PI3K/Akt signalling pathway downstream of this. Upon miR-92b-3p overexpression in the HUVECs using SKOV3-92b/exo, qRT-PCR and Western blot analyses revealed decreased mRNA and protein levels of SOX4 and ET-1, as well as a reduction in phosphorylated AKT protein levels [64]. This SOX4 inhibition, and consequently ET-1 and phosphorylated AKT repression, resulted in reduced tube formation and hindered the migration of the HUVECs. HUVECs treated with miR-92b-3p mimics and Apatinib (a tyrosine kinase inhibitor targeting VEGFR2) exhibited reduced expression of SOX4, ET-1 and phosphorylated AKT compared to those treated with miR-92b-3p mimics or Apatinib alone [64]. The authors engineered SKOV3-92b/exo with peptides to improve delivery to the blood vessels (Arg-Gly-Asp). In vivo application of these modified exosomes to nude mice saw a significant inhibition in tumour growth and metastasis, in the presence of and without Apatinib [64]. This highlights the opportunity to use miR-92b-3p to regulate ET-1 expression and target angiogenesis in ovarian cancer cells through the enrichment of exosomes.

Modifying extracellular vesicles to target the ET-1 axis has also been demonstrated within diseases other than cancer, highlighting the potential for this technique to be used in targeting cancer pathways. One example of this is by targeting EDNRA expression in deep vein thrombosis (DVT) using miR-342-3p [65]. In rat models with DVT, decreased expression of miR-342-3p was exhibited, coinciding with increased EDNRA expression. Overexpressing miR-342-3p in human umbilical cord mesenchymal stem cell (hucMSC)-derived exosomes reduced EDNRA expression and improved the disease state of DVT in rat models and restored physiological angiogenesis in HUVECs [65]. This highlights where efforts have been made using EVs to target ET-1 expression, and these preclinical data indicate there to be a therapeutic opportunity to use this method. Thus, further research into this would be interesting so that this technique could be applied in the treatment of various cancers.

There is evidence of success in the targeting of ET-1 in cancer within several in vitro studies, as well as in vivo mice models. Although this has been tested in mice models, the use of ET-1 antagonists in cancer should be reproduced and tested further in ex vivo models. Further research harnessing primary human tumours to be tested within in vivo mice models would also be beneficial. Another interesting point of research would be targeting the ET-1 signalling in primary tumour stem cells. These can also be referred to as cancer stem cells (CSCs), which have the ability to drive tumorigenesis due to their capacity to self-renew and differentiate into multiple lineages. These cells can be induced by differentiated cancer cells and often express drug resistance [66]. It would be interesting to investigate whether targeting ET-1 in human cancer also holds the capacity to affect CSCs.

## 6. Concluding Remarks

From this review, it is evident ET-1 is involved in a complex signalling network, promoting a malignant phenotype according to various pathways and mechanisms. Therapeutic intervention into ET-1 seems to be a promising area of research in an oncological setting, whether that be through the repurposing of ET-1 receptor antagonists or alternative small molecules such as metformin, preventing ET-1 signalling. Many efforts to target this peptide appear to have the focus of targeting ET-1 at the mRNA level or through its receptors. ET-1 has a very short half-life in vivo, potentially making the actual molecule itself difficult to target. Therefore, it could be suggested that intervention may be most successful if targeted at the transcriptional level or by preventing its signalling. ET-1 receptor antagonists have shown promise preclinically in targeting and improving oncogenic pathways in many different cancer types, as discussed throughout this review. However, clinically, these drugs have shown few positive effects, suggesting there is a need to deepen the knowledge surrounding this vasoconstrictor in order to effectively block its malignant effects within cancer.

## Figures and Tables

**Table 1:** Clinical trials targeting ET-1 signalling. NR—not recorded; MTD—maximum tolerated dose; PF—progression-free; N/A—not applicable; *—agonist to ETBR (SP1-1620 is an ET-1 analogue).

Cancer Type	Drug	Target	Phase	Status	Completion Date	Reported Findings	Clinical Trial Number
Prostate	Zibotentan	ETAR	3	Completed	August 2011	No OS improvement No safety concerns	NCT00554229
	Zibotentan with Docetaxel	ETAR	3	Completed	July 2011	No OS improvement	NCT00617669
	Atrasentan	ETAR/ETBR	3	Completed	August 2006	No delay in disease progression Well tolerated	NCT00036543
			3	Completed	June 2007	NR	NCT00046943
			3	Completed	August 2007	NR	NCT00036556
	S0421, Docetaxel and Prednisone with or without Atrasentan	ETAR/ETBR	3	Completed	February 2016	No OS improvement Toxicity issues	NCT00134056
Kidney	Astrasentan	ETAR/ETBR	2	Completed	April 2006	Well tolerated PF projections not met	NCT00039429
Biliary	SPI-1620 with Docetaxel	ETBR *	2	Terminated	September 2015	Toxicity issues	NCT01773785
Glioblastoma	Atrasentan	ETAR/ETBR	1	Completed	August 2008	Determined MTD as 70 mg/day	NCT00017264
	Macitentan with Temozolomide (TMZ)	ETAR/ETBR	1	Completed	April 2016	Well tolerated	NCT01499251
Pancreatic	Bosentan with Gemcitabine and Nab-paclitaxel	ETAR/ETBR	1	Ongoing	N/A	N/A	NCT04158635
Melanoma	BQ-788	ETBR	Early phase 1	Terminated	May 2012	Well tolerated Indicated to directly affect melanoma cell viability	NCT02442466
	Bosentan with Dacarbazine	ETAR/ETBR	2	Completed	February 2008	No effect on time to tumour progression No safety issues	NCT01009177
Solid tumours: ovarian, pancreatic, melanoma	ENB003 with Pembrolizumab	ETBR	1b/2a	Ongoing	N/A	N/A	NCT04205227
Ovarian, fallopian and peritoneal serous adenocarcinoma	Atrasentan with DOXIL	ETAR/ETBR	2	Terminated	March 2009	NR	NCT00653328

## Data Availability

No new data were created or analysed in this study. Data sharing is not applicable to this article.

## References

[1] Jemal A., Bray F., Center M.M., Ferlay J., Ward E., Forman D. (2011). Global cancer statistics. CA Cancer J. Clin..

[2] Sung H., Ferlay J., Siegel R.L., Laversanne M., Soerjomataram I., Jemal A., Bray F. (2021). Global Cancer Statistics 2020: GLOBOCAN Estimates of Incidence and Mortality Worldwide for 36 Cancers in 185 Countries. CA Cancer J. Clin..

[3] Zhao J., Xu L., Sun J., Song M., Wang L., Yuan S., Zhu Y., Wan Z., Larsson S., Tsilidis K. (2023). Global trends in incidence, death, burden and risk factors of early-onset cancer from 1990 to 2019. BMJ Oncol..

[4] Mansoori B., Mohammadi A., Davudian S., Shirjang S., Baradaran B. (2017). The Different Mechanisms of Cancer Drug Resistance: A Brief Review. Adv. Pharm. Bull..

[5] Vasan N., Baselga J., Hyman D.M. (2019). A view on drug resistance in cancer. Nature.

[6] Yanagisawa M., Kurihara H., Kimura S., Tomobe Y., Kobayashi M., Mitsui Y., Yazaki Y., Goto K., Masaki T. (1988). A novel potent vasoconstrictor peptide produced by vascular endothelial cells. Nature.

[7] Rosano L., Spinella F., Bagnato A. (2013). Endothelin 1 in cancer: Biological implications and therapeutic opportunities. Nat. Rev. Cancer.

[8] Banecki K., Dora K.A. (2023). Endothelin-1 in Health and Disease. Int. J. Mol. Sci..

[9] Zhang Z.Y., Chen L.L., Xu W., Sigdel K., Jiang X.T. (2017). Effects of silencing endothelin-1 on invasion and vascular formation in lung cancer. Oncol. Lett..

[10] Kido T., Sawamura T., Masaki T. (1998). The Processing Pathway of Endothelin-1 Production. J. Cardiovasc. Pharmacol..

[11] Boutin G., Yuzugulen J., Pranjol M.Z.I. (2023). Endothelin-based markers for endothelial dysfunction in chemotherapy-induced cardiotoxicity. J. Mol. Cell. Cardiol. Plus.

[12] Liakou P., Tepetes K., Germenis A., Leventaki V., Atsaves V., Patsouris E., Roidis N., Hatzitheophilou K., Rassidakis G.Z. (2012). Expression patterns of endothelin-1 and its receptors in colorectal cancer. J. Surg. Oncol..

[13] Motte S., McEntee K., Naeije R. (2006). Endothelin receptor antagonists. Pharmacol. Ther..

[14] Bagnato A., Salani D., Di Castro V., Wu-Wong J.R., Tecce R., Nicotra M.R., Venuti A., Natali P.G. (1999). Expression of Endothelin 1 and Endothelin A Receptor in Ovarian Carcinoma: Evidence for an Autocrine Role in Tumor Growth1. Cancer Res..

[15] Ma L., Pei G. (2007). Beta-arrestin signaling and regulation of transcription. J. Cell Sci..

[16] Sobolesky P.M., Moussa O. (2013). The role of beta-arrestins in cancer. Prog. Mol. Biol. Transl. Sci..

[17] Tocci P., Blandino G., Bagnato A. (2021). YAP and endothelin-1 signaling: An emerging alliance in cancer. J. Exp. Clin. Cancer Res..

[18] Tocci P., Rosano L., Bagnato A. (2019). Targeting Endothelin-1 Receptor/beta-Arrestin-1 Axis in Ovarian Cancer: From Basic Research to a Therapeutic Approach. Front. Endocrinol..

[19] Rosano L., Cianfrocca R., Tocci P., Spinella F., Di Castro V., Caprara V., Semprucci E., Ferrandina G., Natali P.G., Bagnato A. (2014). Endothelin A receptor/beta-arrestin signaling to the Wnt pathway renders ovarian cancer cells resistant to chemotherapy. Cancer Res..

[20] Tocci P., Roman C., Sestito R., Di Castro V., Sacconi A., Molineris I., Paolini F., Carosi M., Tonon G., Blandino G. (2023). Targeting tumor-stroma communication by blocking endothelin-1 receptors sensitizes high-grade serous ovarian cancer to PARP inhibition. Cell Death Dis..

[21] Wang Z., Liu P., Zhou X., Wang T., Feng X., Sun Y.-P., Xiong Y., Yuan H.-X., Guan K.-L. (2017). Endothelin Promotes Colorectal Tumorigenesis by Activating YAP/TAZ. Cancer Res..

[22] Abylkassov R., Xie Y. (2016). Role of Yes-associated protein in cancer: An update. Oncol. Lett..

[23] Krupska I., Bruford E.A., Chaqour B. (2015). Eyeing the Cyr61/CTGF/NOV (CCN) group of genes in development and diseases: Highlights of their structural likenesses and functional dissimilarities. Hum. Genom..

[24] Masi I., Caprara V., Spadaro F., Chellini L., Sestito R., Zancla A., Rainer A., Bagnato A., Rosano L. (2021). Endothelin-1 drives invadopodia and interaction with mesothelial cells through ILK. Cell Rep..

[25] Semprucci E., Tocci P., Cianfrocca R., Sestito R., Caprara V., Veglione M., Castro V.D., Spadaro F., Ferrandina G., Bagnato A. (2016). Endothelin A receptor drives invadopodia function and cell motility through the beta-arrestin/PDZ-RhoGEF pathway in ovarian carcinoma. Oncogene.

[26] Sestito R., Tocci P., Roman C., Di Castro V., Bagnato A. (2022). Functional interaction between endothelin-1 and ZEB1/YAP signaling regulates cellular plasticity and metastasis in high-grade serous ovarian cancer. J. Exp. Clin. Cancer Res..

[27] Guise T.A., Yin J.J., Mohammad K.S. (2003). Role of endothelin-1 in osteoblastic bone metastases. Cancer.

[28] Said N., Smith S., Sanchez-Carbayo M., Theodorescu D. (2011). Tumor endothelin-1 enhances metastatic colonization of the lung in mouse xenograft models of bladder cancer. J. Clin. Investig..

[29] Wu M.H., Lo J.F., Kuo C.H., Lin J.A., Lin Y.M., Chen L.M., Tsai F.J., Tsai C.H., Huang C.Y., Tang C.H. (2012). Endothelin-1 promotes MMP-13 production and migration in human chondrosarcoma cells through FAK/PI3K/Akt/mTOR pathways. J. Cell. Physiol..

[30] Nie S., Zhou J., Bai F., Jiang B., Chen J., Zhou J. (2014). Role of endothelin A receptor in colon cancer metastasis: In vitro and in vivo evidence. Mol. Carcinog..

[31] Shi L., Zhou S.S., Chen W.B., Xu L. (2017). Functions of endothelin-1 in apoptosis and migration in hepatocellular carcinoma. Exp. Ther. Med..

[32] Sestito R., Cianfrocca R., Tocci P., Rosano L., Sacconi A., Blandino G., Bagnato A. (2020). Targeting endothelin 1 receptor-miR-200b/c-ZEB1 circuitry blunts metastatic progression in ovarian cancer. Commun. Biol..

[33] Berabez R., Routier S., Benedetti H., Ple K., Vallee B. (2022). LIM Kinases, Promising but Reluctant Therapeutic Targets: Chemistry and Preclinical Validation In Vivo. Cells.

[34] Nishida N., Yano H., Nishida T., Kamura T., Kojiro M. (2006). Angiogenesis in Cancer. Vasc. Health Risk Manag..

[35] Grant K., Loizidou M., Taylor I. (2003). Endothelin-1: A multifunctional molecule in cancer. Br. J. Cancer.

[36] Jin X., Dai L., Ma Y., Wang J., Liu Z. (2020). Implications of HIF-1alpha in the tumorigenesis and progression of pancreatic cancer. Cancer Cell Int..

[37] Salani D., Di Castro V., Nicotra M.R., Rosano L., Tecce R., Venuti A., Natali P.G., Bagnato A. (2000). Role of endothelin-1 in neovascularization of ovarian carcinoma. Am. J. Pathol..

[38] Liu J., Wang H., Zhang M., Li Y., Wang R., Chen H., Wang B., Gao X., Song S., Wang Y. (2023). Metformin and simvastatin synergistically suppress endothelin 1-induced hypoxia and angiogenesis in multiple cancer types. Cancer Sci..

[39] Bagnato A., Tecce R., Moretti C., Di Castro V., Spergel D., Catt K.J. (1995). Autocrine actions of endothelin-1 as a growth factor in human ovarian carcinoma cells. Clin. Cancer Res..

[40] Rodas F., Vidal-Vidal J.A., Herrera D., Brown-Brown D.A., Vera D., Veliz J., Puschel P., Erices J.I., Sanchez Hinojosa V., Tapia J.C. (2023). Targeting the Endothelin-1 pathway to reduce invasion and chemoresistance in gallbladder cancer cells. Cancer Cell Int..

[41] Ahn H.M., Kim D.G., Kim Y.J. (2020). Blockade of endothelin receptor A enhances the therapeutic efficacy of gemcitabine in pancreatic cancer cells. Biochem. Biophys. Res. Commun..

[42] Englinger B., Lötsch D., Pirker C., Mohr T., van Schoonhoven S., Boidol B., Lardeau C.-H., Spitzwieser M., Szabó P., Heffeter P. (2016). Acquired nintedanib resistance in FGFR1-driven small cell lung cancer: Role of endothelin-A receptor-activated ABCB1 expression. Oncotarget.

[43] Pushpakom S., Iorio F., Eyers P.A., Escott K.J., Hopper S., Wells A., Doig A., Guilliams T., Latimer J., McNamee C. (2019). Drug repurposing: Progress, challenges and recommendations. Nat. Rev. Drug Discov..

[44] Jamal S., Nagrial A., Joshua A., Eek R., Jamal S. (2020). 803 Phase 1/2 study using ENB-003, a first-in-class selective ETBRi, in combination with pembrolizumab in subjects with advanced refractory solid tumors. J. Immunother. Cancer.

[45] Rajeshkumar N.V., Matwyshyn G., Gulati A. (2007). IRL-1620, a tumor selective vasodilator, augments the uptake and efficacy of chemotherapeutic agents in prostate tumor rats. Prostate.

[46] Rajeshkumar N.V., Rai A., Gulati A. (2005). Endothelin B receptor agonist, IRL 1620, enhances the anti-tumor efficacy of paclitaxel in breast tumor rats. Breast Cancer Res. Treat..

[47] Kim R., Chiorean E.G., Amin M., Rocha-Lima C.M.S., Gandhi J., Harris W.P., Song T., Portnoy D. (2017). Phase 2 study of combination SPI-1620 with docetaxel as second-line advanced biliary tract cancer treatment. Br. J. Cancer.

[48] Feng C., Chen B., Fan R., Zou B., Han B., Guo G. (2023). Polyphenol-Based Nanosystems for Next-Generation Cancer Therapy: Multifunctionality, Design, and Challenges. Macromol. Biosci..

[49] Barinda A.J., Arozal W., Sandhiutami N.M.D., Louisa M., Arfian N., Sandora N., Yusuf M. (2022). Curcumin Prevents Epithelial-to Mesenchymal Transition-Mediated Ovarian Cancer Progression through NRF2/ETBR/ET-1 Axis and Preserves Mitochondria Biogenesis in Kidney after Cisplatin Administration. Adv. Pharm. Bull..

[50] Spinella F., Rosanò L., Decandia S., Di Castro V., Albini A., Elia G., Natali P.G., Bagnato A. (2006). Antitumor Effect of Green Tea Polyphenol Epigallocatechin-3-Gallate in Ovarian Carcinoma Cells: Evidence for the Endothelin-1 as a Potential Target. Exp. Biol. Med..

[51] Aliabadi P., Sadri M., Siri G., Ebrahimzadeh F., Yazdani Y., Gusarov A.M., Kharkouei S.A., Asadi F., Adili A., Mardi A. (2022). Restoration of miR-648 overcomes 5-FU-resistance through targeting ET-1 in gastric cancer cells in-vitro. Pathol. Res. Pract..

[52] Xu P., Wu Q., Yu J., Rao Y., Kou Z., Fang G., Shi X., Liu W., Han H. (2020). A Systematic Way to Infer the Regulation Relations of miRNAs on Target Genes and Critical miRNAs in Cancers. Front. Genet..

[53] Li D., Yang P., Li H., Cheng P., Zhang L., Wei D., Su X., Peng J., Gao H., Tan Y. (2012). MicroRNA-1 inhibits proliferation of hepatocarcinoma cells by targeting endothelin-1. Life Sci..

[54] Zmarzly N., Januszyk S., Mieszczanski P., Morawiec E., Buda P., Dziobek K., Oplawski M., Boron D. (2023). Endothelin-3 is epigenetically silenced in endometrioid endometrial cancer. J. Cancer Res. Clin. Oncol..

[55] Lu J., Zhao F.P., Peng Z., Zhang M.W., Lin S.X., Liang B.J., Zhang B., Liu X., Wang L., Li G. (2014). EZH2 promotes angiogenesis through inhibition of miR-1/Endothelin-1 axis in nasopharyngeal carcinoma. Oncotarget.

[56] Hu X., Liu H., Li C. (2023). MiRNA-19b-3p downregulates the endothelin B receptor in gastric cancer cells to prevent angiogenesis and proliferation. Acta Biochim. Pol..

[57] Sestito R., Cianfrocca R., Rosano L., Tocci P., Semprucci E., Di Castro V., Caprara V., Ferrandina G., Sacconi A., Blandino G. (2016). miR-30a inhibits endothelin A receptor and chemoresistance in ovarian carcinoma. Oncotarget.

[58] Kalles V., Zografos G.C., Provatopoulou X., Kalogera E., Liakou P., Georgiou G., Sagkriotis A., Nonni A., Gounaris A. (2012). Circulating levels of endothelin-1 (ET-1) and its precursor (Big ET-1) in breast cancer early diagnosis. Tumor Biol..

[59] Yuzugulen J., Douthwaite J.A., Wood E.G., Villar I.C., Patel N.S.A., Jegard J., Gaertner H., Rossitto-Borlat I., Rose K., Hartley O. (2017). Characterisation of preproendothelin-1 derived peptides identifies Endothelin-Like Domain Peptide as a modulator of Endothelin-1. Sci. Rep..

[60] Irfan S., Zaidi N., Tiwari K., Lal N., Srivastava A.N., Singh S. (2023). Evaluation of salivary endothelin-1 as a biomarker for oral cancer and precancer. J. Cancer Res. Ther..

[61] Golinelli G., Mastrolia I., Aramini B., Masciale V., Pinelli M., Pacchioni L., Casari G., Dall’Ora M., Soares M.B.P., Damasceno P.K.F. (2020). Arming Mesenchymal Stromal/Stem Cells Against Cancer: Has the Time Come?. Front. Pharmacol..

[62] Chulpanova D.S., Kitaeva K.V., Tazetdinova L.G., James V., Rizvanov A.A., Solovyeva V.V. (2018). Application of Mesenchymal Stem Cells for Therapeutic Agent Delivery in Anti-tumor Treatment. Front. Pharmacol..

[63] Vieira J.M.F., Zamproni L.N., Wendt C.H.C., Rocha de Miranda K., Lindoso R.S., Won Han S. (2022). Overexpression of mir-135b and mir-210 in mesenchymal stromal cells for the enrichment of extracellular vesicles with angiogenic factors. PLoS ONE.

[64] Wang J., Wang C., Li Y., Li M., Zhu T., Shen Z., Wang H., Lv W., Wang X., Cheng X. (2021). Potential of peptide-engineered exosomes with overexpressed miR-92b-3p in anti-angiogenic therapy of ovarian cancer. Clin. Transl. Med..

[65] Pan Z., Chen Q., Ding H., Li H. (2022). MicroRNA-342-3p loaded by human umbilical cord mesenchymal stem cells-derived exosomes attenuates deep vein thrombosis by downregulating EDNRA. J. Thromb. Thrombolysis.

[66] Ayob A.Z., Ramasamy T.S. (2018). Cancer stem cells as key drivers of tumour progression. J. Biomed. Sci..

